# Supplementary material for: Root Exudates Alter the Expression of Diverse Metabolic, Transport, Regulatory, and Stress Response Genes in Rhizosphere Pseudomonas
Source: Front Microbiol. 2021 Apr 14;12:651282. doi: 10.3389/fmicb.2021.651282 (PMC8079746; doi:10.3389/fmicb.2021.651282)
Supplement: Supplementary file 2 [file Data_Sheet_1.PDF]

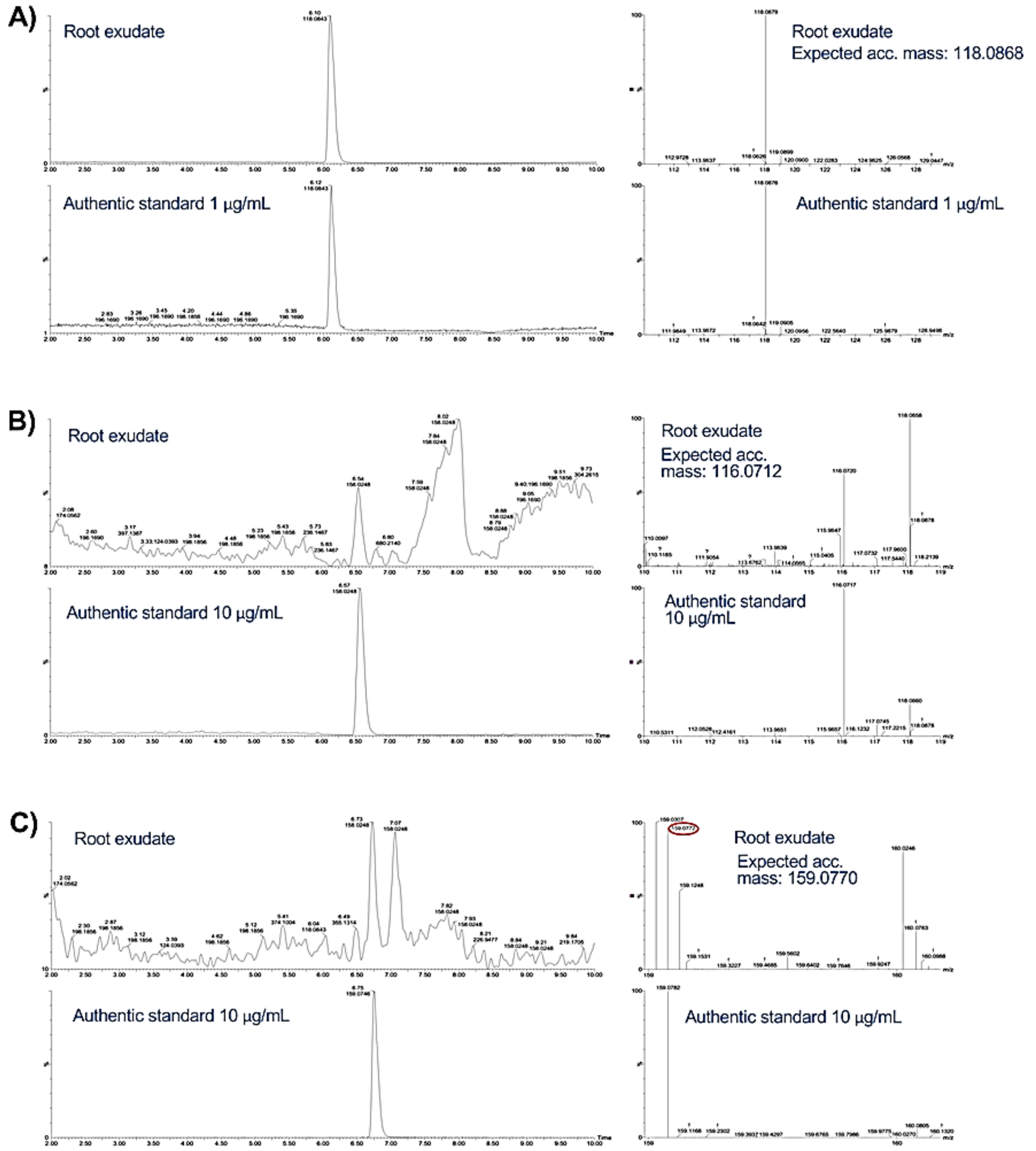

Figure S1. Mass chromatograms of root secretions and authentic standards showing the presence of glycine betaine (A), proline (B), and hydroxyectoine (C) in exudates of hydroponically grown *Brachypodium* plants. For each compound, panels on the left show selected ion chromatograms, while panels on the right depict accurate mass comparisons.

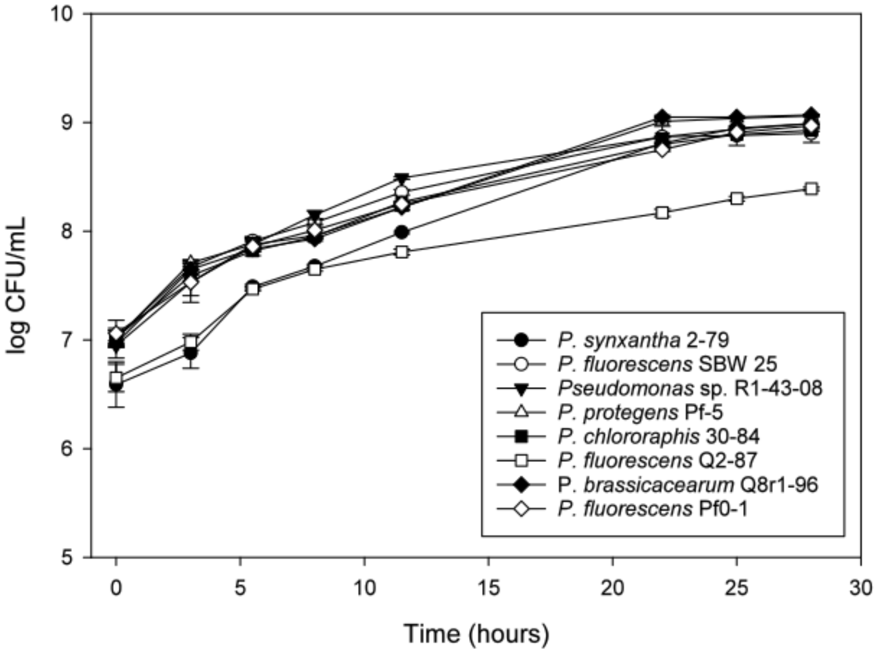

Figure S2. The growth of the studied *Pseudomonas* strains at 25°C in 21C medium amended with root exudates. To mimic rhizosphere conditions, the bacteria were grown statically at 72% air saturation.

Figure S3. Metabolic map of upregulated pathways.

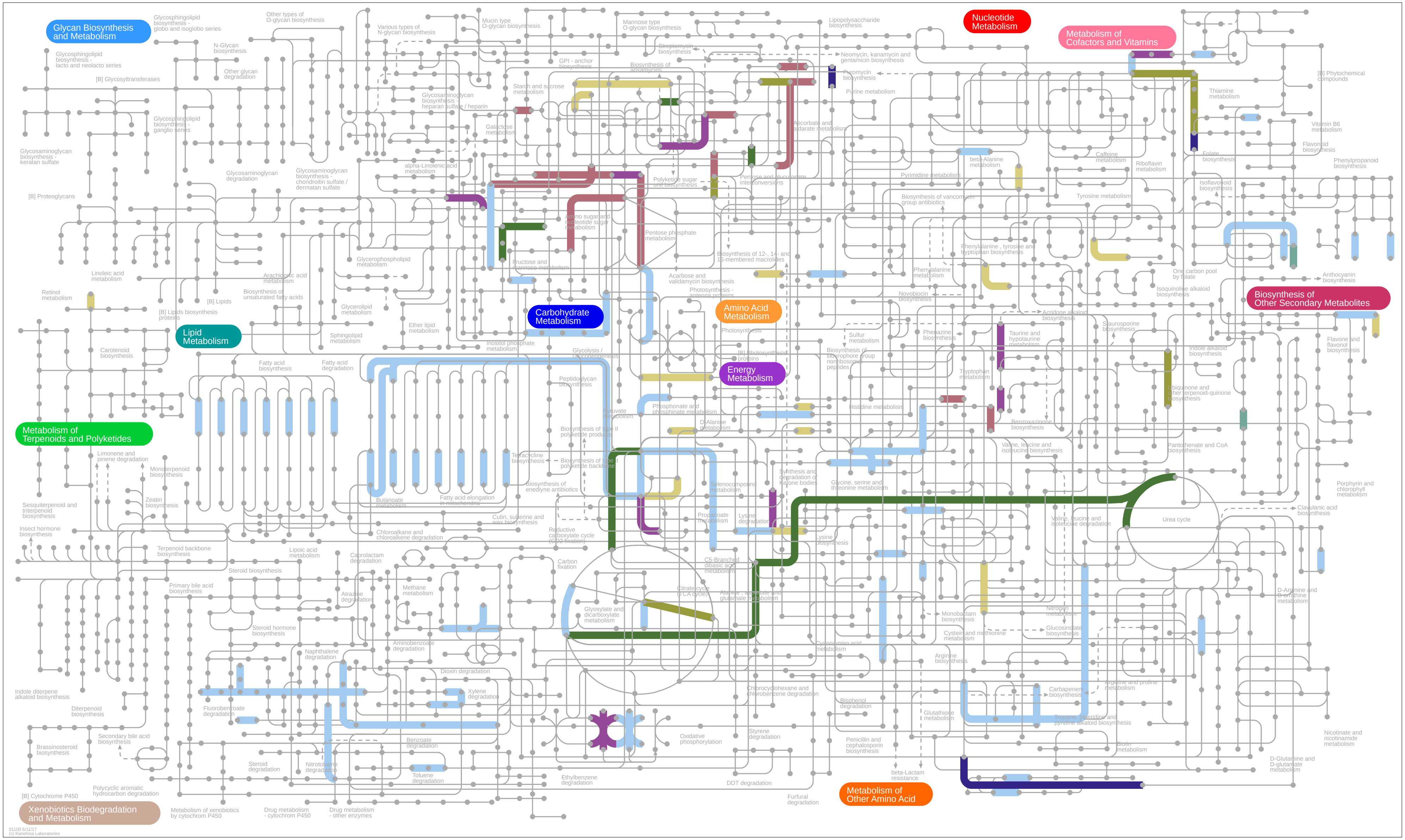

- *P. synxantha* 2-79
- *P. fluorescens* SBW25
- *Pseudomonas* sp. R1-43-08
- *P. brassicacearum* Q8r1-96
- *P. fluorescens* Q2-87
- *P. chlororaphis* 30-84
- *P. protegens* Pf-5
- *P. fluorescens* Pf0-1

Figure S4. Metabolic map of downregulated pathways.

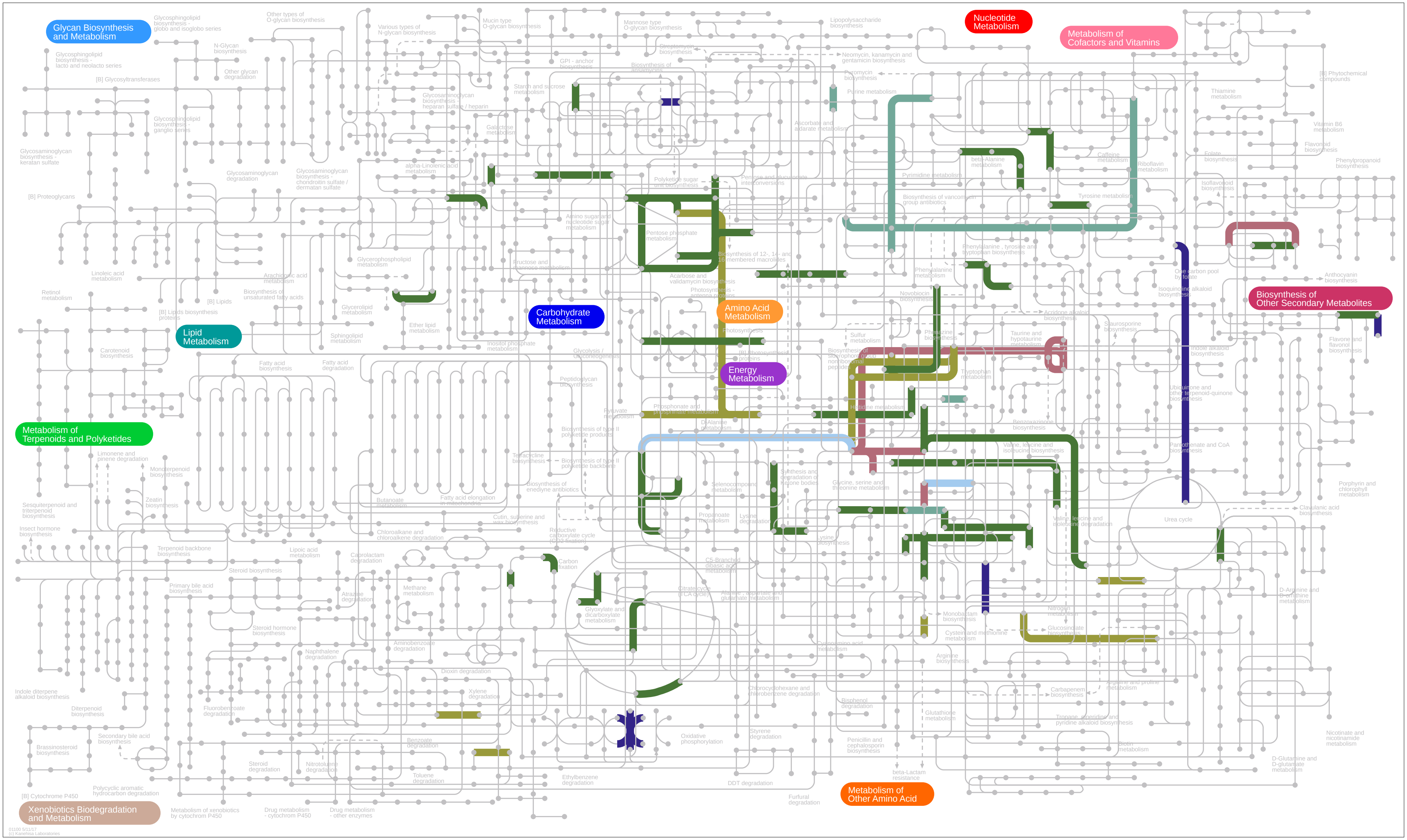

- *P. synxantha* 2-79
- *P. fluorescens* SBW25
- *Pseudomonas* sp. R1-43-08
- *P. brassicacearum* Q8r1-96
- *P. fluorescens* Q2-87
- *P. chlororaphis* 30-84
- *P. protegens* Pf-5
- *P. fluorescens* Pf0-1

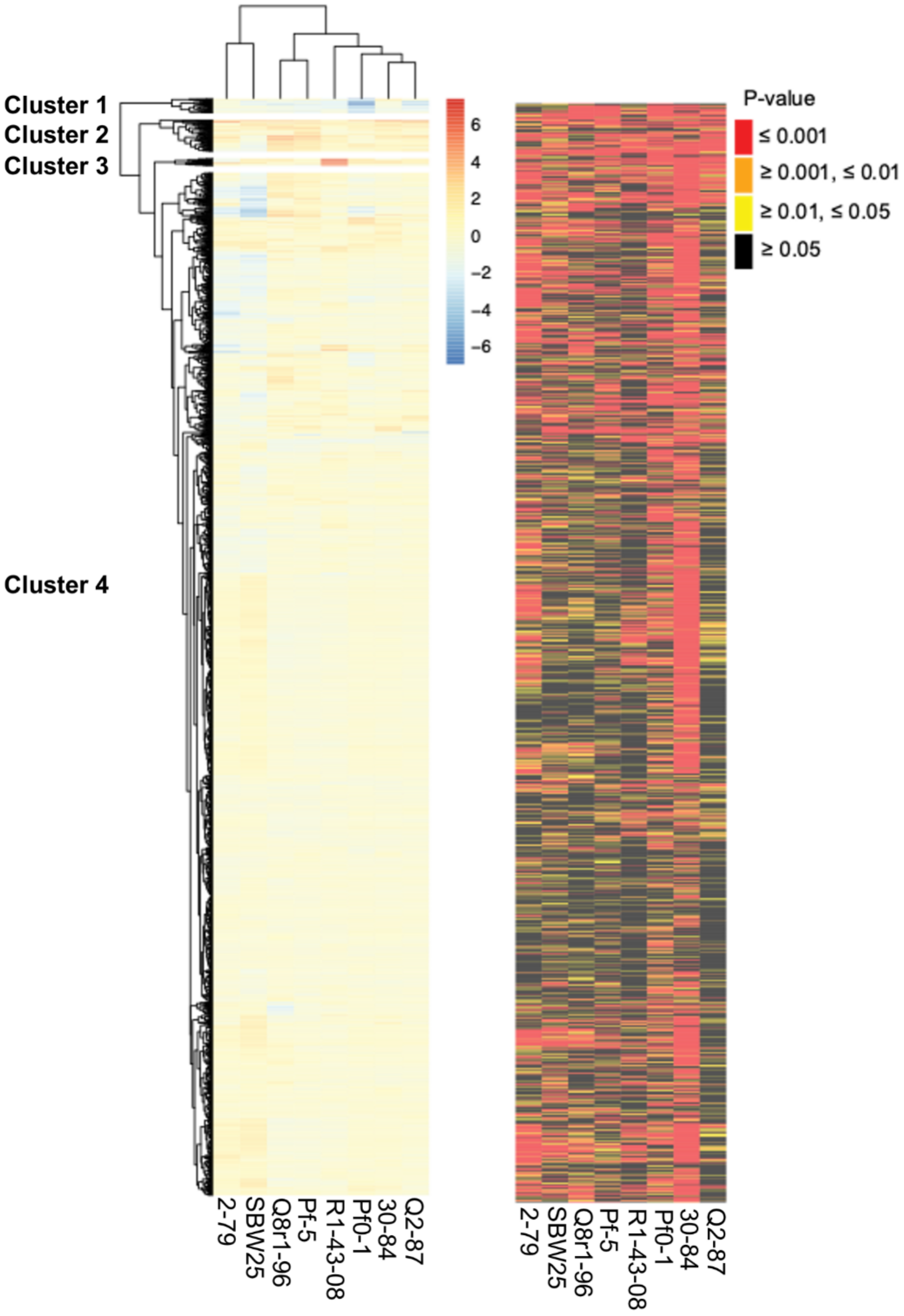

Figure S5. Hierarchical clustering heatmap for core *Pseudomonas* genes (left panel) constructed using Euclidean distance. The clustering was computed using the hclust function of the pheatmap R module (https://CRAN.R-project.org/package=pheatmap). Also shown is the heatmap of corresponding p-adj values (right panel). The corresponding numerical data are listed in supplemental table S13.
